# Adaptive User Interfaces for People with Cognitive Disabilities within the Easy Reading Framework

**DOI:** 10.1007/978-3-030-58805-2_7

**Published:** 2020-08-12

**Authors:** Peter Heumader, Klaus Miesenberger, Tomas Murillo-Morales

**Affiliations:** 8 Institute Integriert Studieren, JKU Linz, Linz, Austria; 9 Jack Baskin School of Engineering, UC Santa Cruz, Santa Cruz, CA USA; 10 Dipartimento di Meccanica, Politecnico di Milano, Milan, Italy; 11 Support Centre for Students with Special Needs, Masaryk University Brno, Brno, Czech Republic; Institut Integriert Studieren, Johannes Kepler Universität Linz, Altenbergerstraße 69, 4040 Linz, Austria

**Keywords:** Cognitive accessibility, Adaptive user interfaces, Web-accessibility

## Abstract

Adaptive user interfaces are user interfaces that dynamically adapt to the users’ preferences and abilities. These user interfaces have great potential to improve accessibility of user interfaces for people with cognitive disabilities. However automatic changes to user interfaces driven by adaptivity are also in contradiction to accessibility guidelines, as consistence of user interfaces is of utmost importance for people with cognitive disabilities. This paper describes how such user interfaces are implemented within the Easy Reading framework, a framework to improve the accessibility of web-pages for people with cognitive disabilities.

## Introduction

The concept of user interfaces that have the ability to change according to the user’s requirements, skills, environment, situation, or other criteria has been around for a long time. In general, these concepts can be categorized in adaptive user interfaces and adaptable user interfaces.

- Adaptive User Interfaces [1]: These systems change their structure, functionalities, and content for the individual user in real time. This is achieved by monitoring the user status, the system state, and the current situation that the user is facing. By using an adaption strategy (mostly rule based), the user interface is changed at run time.
- Adaptable User Interfaces [2]: This user interfaces are highly adjustable in terms of presentation of information, display of user interface and its components or user interaction/input concepts. The settings are usually stored in a user profile and the user is able to adjust those settings in advance, usually in a settings dialog. During runtime, in contrary to the adaptive user interfaces, these settings do not change.

According to Laive [3], methods for user interface adaptations can further be assigned to the following categories:

- Adaptable/Manual: the user manages the process and performs all actions
- Adaptable with system support/user selection: the user dominates the adaptation process and the system supports it
- Adaptive with user control/user approval: the system dominates the adaptation process under the supervision of the user. The system initiates the action and notifies the user about the alternative that he/she has to choose
- Adaptive/Fully adaptive: the whole process is managed by the system, which decides and implements the action based on the preferential model and the main uses

Adaptive user interfaces show great potential towards enhancing the usability and accessibility of computer systems. User tracking with state-of-the-art sensors could give estimations about the current user’s status, and could trigger adequate system reactions based on that [4, 5].

However, the added adaptability for user interfaces to improve accessibility might have some unwanted side effects. For example, increasing the font size to address the vision impairment of a person might result in longer text passages and the need to scroll, which in turn results in increased attention and memory demands for the user. Therefore, providing extensive adaptability is a highly complex task, as side effects and conflicts are difficult to locate [10]. Another unwanted site effect of fully adaptive user interfaces is the inconsistency caused by the dynamic changes to the user profile, which is then reflected in the user interface. This is another drawback, as consistency across webpages is very important for people with cognitive disabilities and also addressed in Guideline 2.1: Predictable of the W3C Web Content Accessibility Guidelines (WCAG2.1) [8, 11].

This paper describes how user interface adaptations are realized within the Easy Reading framework. Easy Reading is a software tool that supports cognitive accessibility of web content and enables people with cognitive disabilities to better read, understand and use web pages. This is achieved through functionalities as:

- Adjustment of the layout and structure of webpages,
- Explanation/Annotation of web content with symbols, videos or pictures,
- Automatic/supported Modification of web content e.g. by translating it into plain language or easy2read.

Easy Reading has been designed as a cloud based solution, allowing people to interact with clients implemented as browser extension or mobile applications. Within the framework, user interfaces, user interaction and the provided help are adaptable and, to a certain extent, also adaptive for the individual user.

## State of the Art

In recent years several research projects have been dealing with the creation of adaptive user interfaces for people with disabilities. Among those projects, prominent examples are GPII [6] or MyUI [7].

GPII allows the personalization of user interfaces, by the use of cross-platform user profiles for user interface settings, and rule-based and statistical approaches for matchmaking [14]. The architecture of the GPII was developed by the Cloud4all project uses an ontology of user needs and preferences that are directly linked to user settings [9]. The linking is done with a rule based matchmaker (RBMM) that matches user preferences and needs, with solutions available to the system and settings supported by the solution. The matchmaker results therefore in a fully configured solution on the specific system based on the individual preferences and needs of a user [6].

MyUI on the other hand was an EU funded project that enabled the generation of individualized user interfaces that would adapt to the individual users needs in real-time, based on a user profile and the actual device [10, 12, 13].

These approaches all work with a user profile that is usually stored online. Once the user logs in, the profile is downloaded and a mechanism uses this profile to create a dynamic configuration of software, assistive technology, user interface or the whole operating system for the individual user. Adaptations can only be made on features and software that are currently available on the actual device or software, and therefore the user experience might change on different devices. While this approach is sufficient for most users, it is problematic for people with cognitive disabilities, as consistency of user interfaces is very important for them [8]. Another drawback of this solution is that features must be installed on the device first, before they can be used and adapted, which might be another obstacle for people with cognitive disabilities.

## Approach

The Easy Reading framework allows users to obtain assistance for difficult to cope with content on any webpage. This is done by cloud based software-clients that inject a dynamically generated user interface directly in the current page. By this users are able to trigger different forms of help provided by the framework. The result of the support is then rendered again directly in the webpage – allowing the user to stay at the original content and learning to cope with it in the future.

**Fig. 1.**
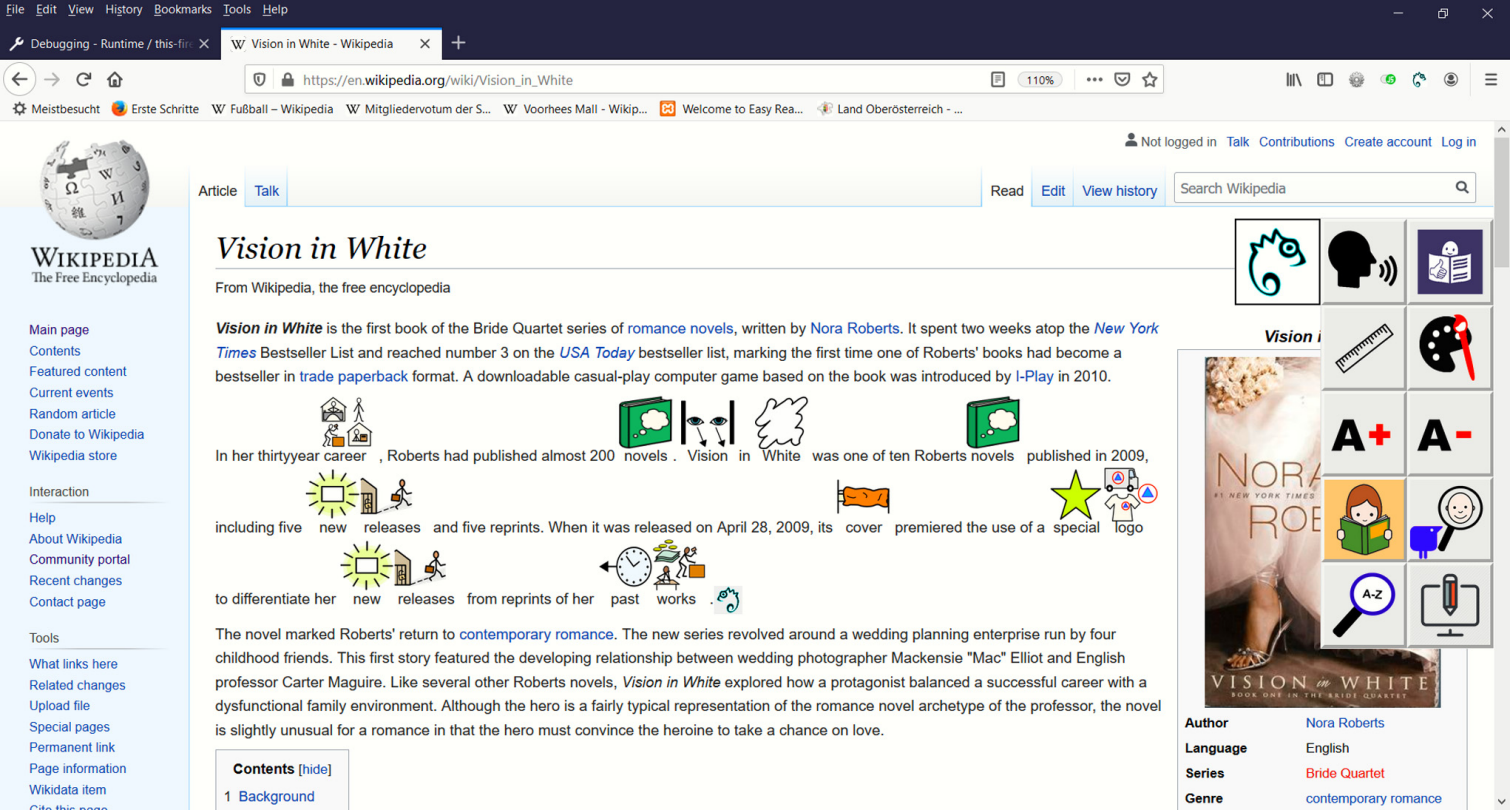
Easy Reading on a webpage

Figure 1 shows a screenshot of Easy Reading on a Wikipedia page. The user interface is dynamically injected on the right – the result of triggering an assistance tool provided by the framework is directly rendered within the web-page. In this case the help was an automatically crated AAC^1^-version of the second paragraph accomplished by a text analysis cloud service in combination with an AAC library.

Adaptations within the Easy Reading framework can be applied to the user interface, the help that is provided, the user interaction (how help is triggered) and finally how the help is rendered and presented within the web-page. Similarly to existing approaches, these adaptations are based on a user profile that stores user preferences and abilities. Currently the user profile hosts the following support categories for the help provided by the framework:

- Text Support: Indicates whether and how the user needs help with text and content in general.
- Layout Support: How the layout of Websites should be displayed for the user.
- Reading Support: If and how the user needs support in reading text
- Symbol Support: Indicates if and how the user needs support with symbol language

In addition, the profile holds categories for triggering and displaying the provided help:

- Input Support: Stores the preferred way to triggering help and to select where on the web-page help is needed
- Output Support: Specifies the preferred way of rendering the help provided

**Fig. 2.**
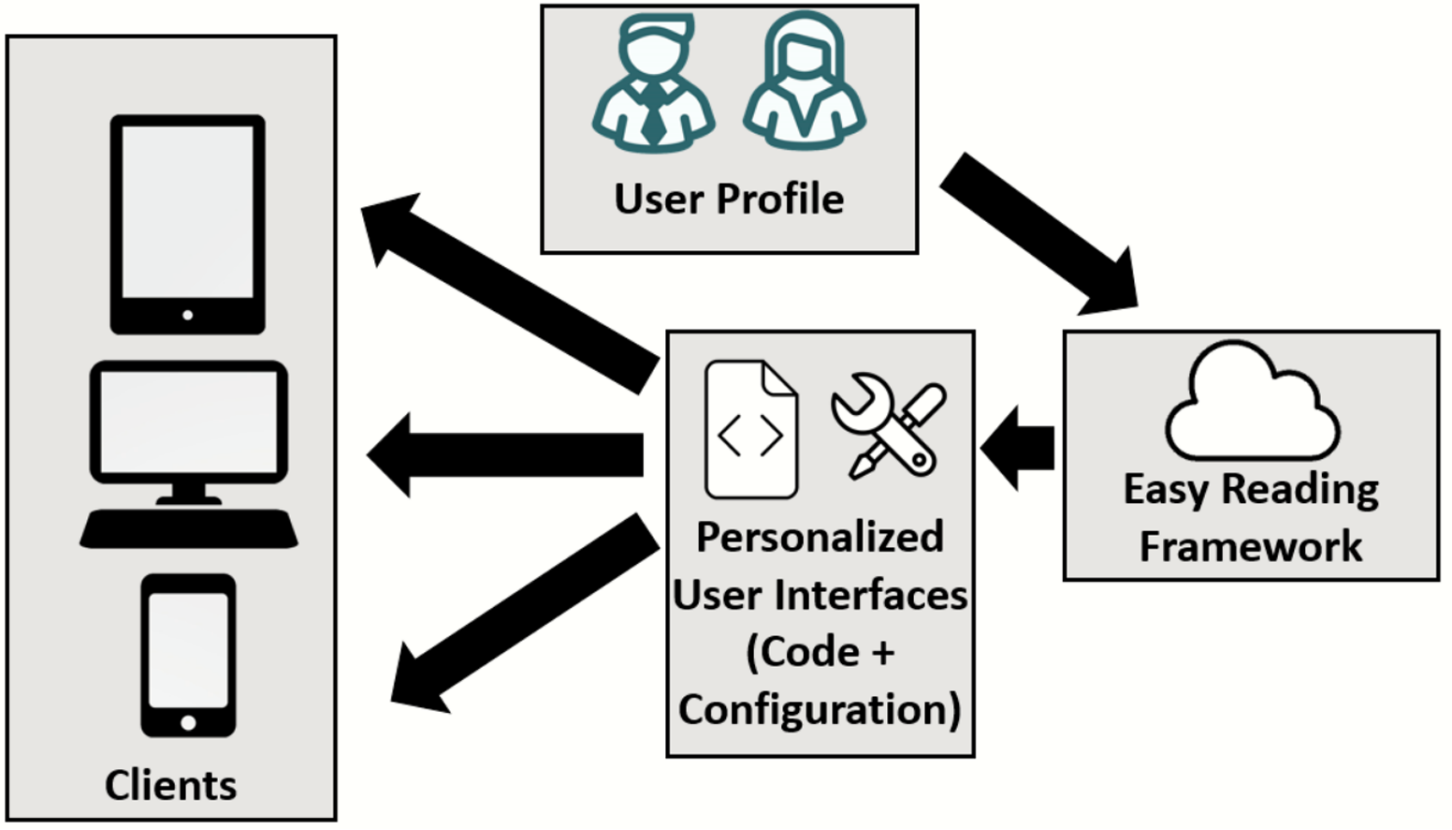
User interfaces dynamically created and configured for the individual user

Based on these categories, once the user logs in with his or her user profile, a dynamically optimized configuration is created for the individual user (see Fig. 2). Unlike other approaches, this configuration is not created locally, but in the cloud, and it also includes personalized user interfaces, personalized help and a personalized way of displaying the help. In this manner, clients within the Easy Reading framework do not host the code for any feature provided by the framework, as this is dynamically created for each user.

This is a big advantage over other architectures, as no matter from which device the user logs in, the user experience is always the same. Another advantage is that no additional software needs to be installed, as every feature is prepared in the cloud and downloaded during user login. Finally this enables learning and improving personalization of service provision cross different web pages and over time.

A drawback of the solution is however that it only works within a browser, while other solutions like GPII would also work across different applications or even operating systems. Here however each application has to be GPII-compliant and must implement its interfaces. Expanding the Easy Reading approach towards this broader application scenarios are considered as future challenges.

## Update Strategies

**Fig. 3.**
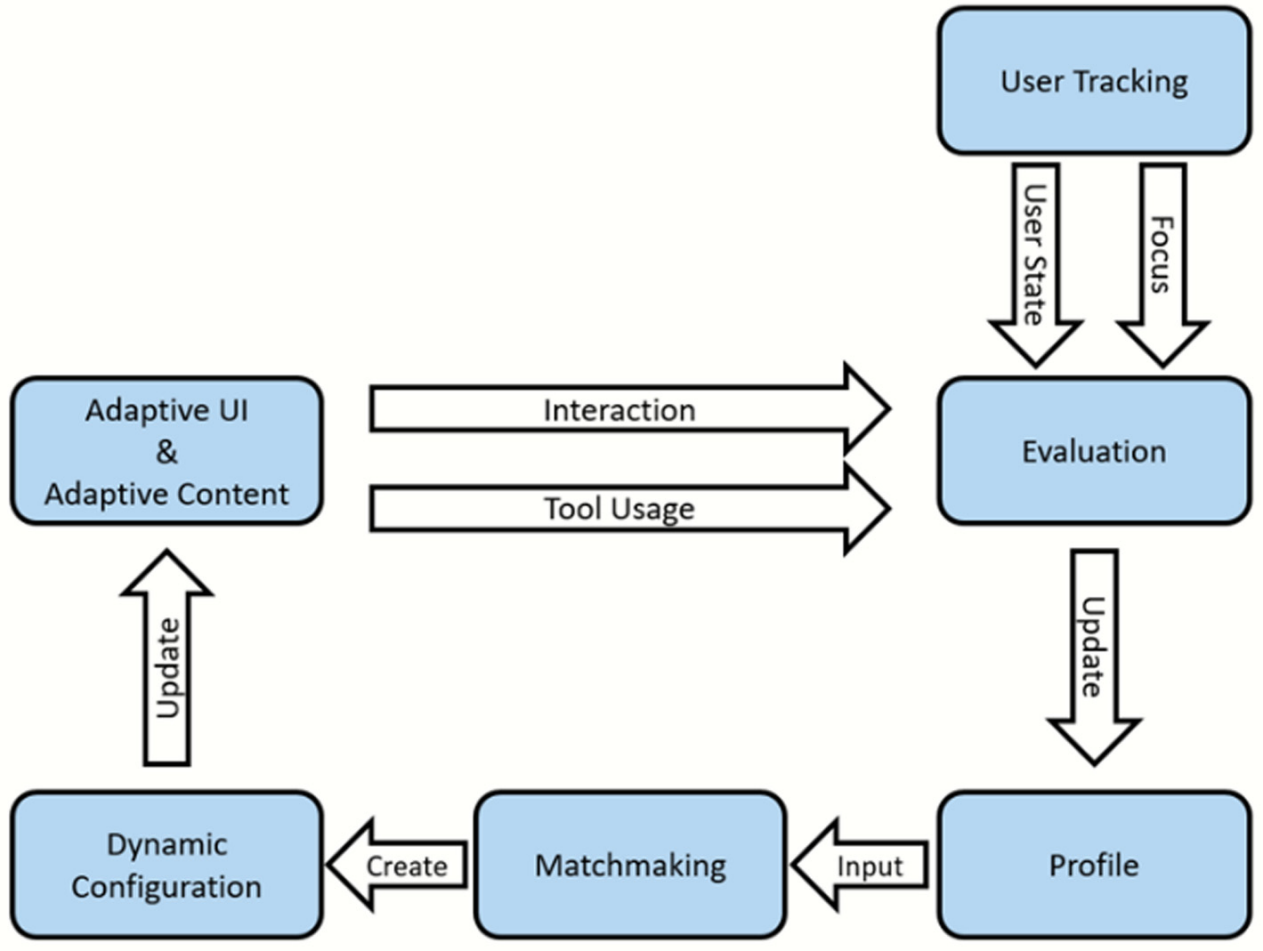
Adaptive workflow within the Easy Reading framework

As user skills, know-how and preferences change over time, the Easy Reading framework hosts a mechanism that automatically updates the preferences of the user profile based on user tracking and usage statistics (Fig. 3).

While the user is surfing the web, user interaction and tool usage is evaluated, and an updated profile is calculated. In addition, the system also hosts an additional optional user tracking component that creates an estimation on the users’ current focus and detects and understands the situation the user faces (e.g. attention, stress, and confusion) at the moment. By this the additional feedback is created whether

- the user needs help for a part of the content,
- the help applied by the framework is accepted by the user
- the user has problems with the user interface or the user interaction required to trigger help of the framework

User tracking combines different sensors that feed into a software reasoner to calculate this estimation. Currently an eye-tracker that tracks the focus of the user on the web-page is used to detect cognitive load. Additionally, a smartwatch that detects heartbeats and heart rate variability is utilized to detect stress.

Based on this sensor data and the user interaction on the web-page, every hour the matchmaking component is triggered with the updated profile, resulting in a new dynamic configuration. Based on this a recommendation to add or remove functionality is triggered and presented via a dialog to the user.

**Fig. 4.**
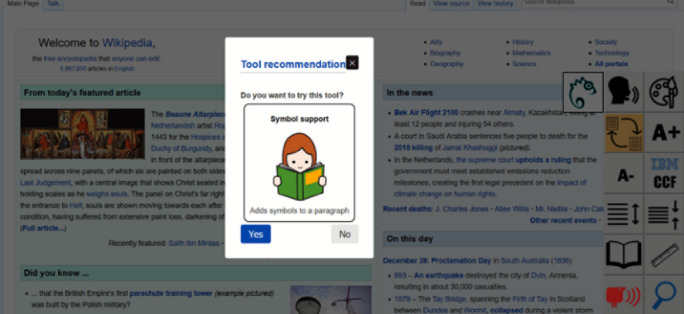
Tool recommendation within the Easy Reading framework

Figure 4 shows such a recommendation dialog. If the user accepts the dialog, the updated profile is saved and the tool is added into the current user interface. On the other hand, if the user rejects the recommendation, the changes to the profile are reverted. User approval of any changes is of utmost importance, as consistency of user interfaces and user interaction must be preserved.

## Current Results and Further Work

Currently the system is able to make recommendations for different tools to simplify web content as well as for different user interfaces that the framework provides. In the future recommendations on changing user interaction to trigger tools and displaying help provided by the framework will be implemented.

Due to the COVID-19 outbreak large scale user tests were not possible. Preliminary tests with 8 end users showed that a purely adaptive user interface without any user approval is not appropriate for end users. On the other hand, most users were able to understand the current implementation with user approval. Once the COVID-19 situation allows it, more exhaustive user tests are planned.

## Footnotes

1 https://en.wikipedia.org/wiki/Augmentative_and_alternative_communication.
